# Growth of Collagen Fibril Seeds from Embryonic Tendon: Fractured Fibril Ends Nucleate New Tip Growth

**DOI:** 10.1016/j.jmb.2010.04.008

**Published:** 2010-05-28

**Authors:** David F. Holmes, Alexander Tait, Nigel W. Hodson, Michael J. Sherratt, Karl E. Kadler

**Affiliations:** 1 Wellcome Trust Centre for Cell-Matrix Research, Faculty of Life Sciences, University of Manchester, Michael Smith Building, Oxford Road, Manchester M13 9PT, UK; 2 Tissue Injury and Repair Group, Faculty of Medical and Human Sciences, University of Manchester, Manchester M13 9PT, UK

**Keywords:** TEM, transmission electron microscopy, STEM, scanning transmission electron microscopy, AFM, atomic force microscopy, collagen fibrils, seeding, surface nucleation, fibril growth, electron microscopy

## Abstract

Collagen fibrils are the principal tensile element of vertebrate tissues where they occur in the extracellular matrix as spatially organised arrays. A major challenge is to understand how the mechanisms of nucleation, growth and remodelling yield fibrils of tissue-specific diameter and length. Here we have developed a seeding system whereby collagen fibrils were isolated from avian embryonic tendon and added to purified collagen solution, in order to characterise fibril surface nucleation and growth mechanisms. Fragmentation of tendon in liquid nitrogen followed by Dounce homogenisation generated fibril length fragments. Most (> 94%) of the fractured ends of fibrils, which show an abrupt square profile, were found to act as nucleation sites for further growth by molecular accretion. The mechanism of this nucleation and growth process was investigated by transmission electron microscopy, atomic force microscopy and scanning transmission electron microscopy mass mapping. Typically, a single growth spur occurred on the N-terminal end of seed fibrils whilst twin spurs frequently formed on the C-terminal end before merging into a single tip projection. The surface nucleation and growth process generated a smoothly tapered tip that achieved maximum diameter when the axial extension reached ∼ 13 μm. Lateral growth also occurred along the entire length of all seed fibrils that contained tip projections. The data support a model of collagen fibril growth in which the broken ends of fibrils are nucleation sites for propagation in opposite axial directions. The observed fibril growth behaviour has direct relevance to tendon matrix remodelling and repair processes that might involve rupture of collagen fibrils.

The initial collagen fibril matrix in embryonic tendon consists of fibrils ∼ 30 nm in diameter. During embryonic development, fibrils grow steadily in diameter such that there is a steady increase in mass per unit length in the chick metatarsal tendon from 12 to 18 days.^1^ Fibril length also increases several fold,^2,3^ but measurements of fibril length are limited by practical difficulties in isolating intact fibrils beyond ∼ 100 μm. Postnatal maturation of tendon involves a further phase of fibril growth (of both diameter and length) over a time scale of several months.^4^ The mechanisms of these controlled growth process are largely unknown. Fibrils can increase in size by both molecular accretion and interfibrillar fusion, but the mechanisms and relative contributions of these controlled growth processes have not been established.

Several models have been proposed for the growth of individual collagen fibrils (e.g., see Refs. 5 and 6) that differ in the nature of the rate-limiting steps for molecular accretion onto the fibril surface. In recent years, precise size and shape data on growing fibrils in tissue and cell-free assembly systems have been obtained with quantitative scanning transmission electron microscopy (STEM) (for a review, see Ref. 7). Studies of tissue fibrils have included the mutable tissues of echinoderms where, in contrast to vertebrate tissues, entire collagen fibrils with lengths of up to 2 mm can be readily isolated. A simple surface nucleation and propagation model^8^ (SNAP) was proposed on the basis of these quantitative STEM data. This model was successful, using a minimum of two independent kinetic parameters, in predicting growth curves that were in agreement with experimental data for echinoderm fibrils.

Several key questions remain unanswered about the mechanism of axial and lateral growth of fibrils in tissue during growth and remodelling. Here, we have developed a simple seeding system in which fragments of tissue fibrils are incubated in a solution of purified type I collagen. The initial results from this system show that tissue fibril fragments with abruptly broken ends act as nuclei and grow in length and diameter by the accretion of type I collagen molecules.

### A seeding system for the growth of collagen fibrils from tissue

Mechanical dispersion of chick embryonic tendon after crushing in liquid nitrogen produced fibril length fragments. Most of these (> 80%) showed two abruptly fractured, blunt ends with no apparent fraying (Fig. 1a). These fibrils were seeded into warm neutral solutions of type I collagen of sufficiently low concentration to prevent significant new fibril nucleation. We were careful to avoid the independent nucleation of new collagen fibrils by first preincubating the diluted collagen at 34 °C for 30 min and then centrifuging the sample to remove any early fibrils that might have formed. Electron microscopy of the supernatant showed the absence of fibrils. In further control experiments, the supernatant was incubated for up to 24 h and then examined by transmission electron microscopy (TEM). Again, in this sample, no significant numbers of fibrils were observed. This method of preclearing any early fibrils was used in all experiments in which seed fibrils were studied.

### Kinetics of nucleation and growth

Growth spurs were seen on the seed fibrils after incubation for 30 min (Fig. 1b) and the number and length of these growth projections increased steadily with incubation time (Fig. 1c–f). After 24 h of incubation, ∼ 94% of the blunt ends of seed fibrils had regrown to form a new tapered tip projection. The new tips appeared to grow steadily by molecular accretion at a rate of ∼ 0.5 μm per hour under the experimental conditions used here (50 μg/ml collagen at 34 °C, *I* 0.2 and pH 7.4). Numbers of seed fibrils with and without growth projections were counted during incubation times of up to 24 h (Fig. 1g). The increase in the proportion of fibril ends containing projections (i.e., spurs) was consistent with an exponential rate loss of seeds. Analysis of the kinetic data showed a nucleation half-time of ∼ 2.1 h per tip. Projection length measurements were also made for a range of incubation times (Fig. 1h). Overall, there was a steady increase in the length of the projection over the incubation period with an ∼ 8-fold increase in mean projection length from 2 to 24 h.

### Mass mapping of tip growth and lateral growth of seed fibrils

STEM mass mapping (Fig. 2) was used to measure the lateral size (mass per unit length) of the fibril projections. Axial mass distributions were measured for a number of fibril tip projections of varying length. A typical STEM image and corresponding axial mass distribution is shown in a and b, respectively, in Fig. 2. The new tips were smoothly tapered and showed a limiting lateral size of ∼ 600 kDa/nm when projections attained a length of ∼ 200 *D* periods (Fig. 2c). These observations raised the question of whether the lateral size of the projection was limited to that of the seed fibril or by the inherent limit in lateral size of “early fibrils” reconstituted from the pure type I collagen solution. Early fibrils refers to fibrils generated at an early stage of reconstitution with both fibril ends visible before interfibrillar fusion occurs.^10^ Measurements of mass per unit length were made on a large sample of fibrils with projections 24 h after incubation (see Fig. 2e). Measurements were made at four key sites (as shown): the midpoint of the seed fibril shaft (A), both sides of the seed-projection junction (B, C) and the point of maximum mass per unit length along the tip projection (D). The mean values of mass per unit length are shown compared with those of the original seed fibrils and the early fibrils formed independently from the collagen solution (200 μg/ml). The results show that the maximum mass per unit length of the projections is ∼ 2 × that of the reconstituted early fibrils and ∼ 1.26 × that of the original seed fibrils. The results also indicate a major lateral growth of the seed fibrils with a 2.6-fold increase in mean mass per unit length after 24 h of growth. Comparison of the mass-per-unit length histograms (Fig. 2d) indicates that this lateral growth occurs for the whole population of seed fibrils.

### Axial structure across the seed–tip interface

In the next series of experiments we wanted to learn more about how collagen molecules accrete onto the blunt ends to generate the new tip projections. We used atomic force microscopy (AFM) and STEM of unstained fibrils and TEM of negatively stained fibrils to study the ultrastructure across the junction between the seed fibril and the new tip. A typical AFM image is shown in Fig. 3a with a straightened fibril + tip projection and corresponding height profile in Fig. 3b. This, along with other AFM images, shows a continuity of the periodic gap–overlap structure across the junction region. This continuity is also observed in STEM images and the derived axial mass distributions (Fig. 3c and d). Analysis of TEM images of negatively stained fibrils showed that the molecular polarity was preserved across the fibril seed–tip junctions (Fig. 3e). Although some fibril ends had one spur (e.g., Fig. 3e), a large proportion (∼ 42% after 24 h) of fibril ends contained two spurs, and in these cases the molecular polarity was preserved in both spurs (Fig. 3f). In twin-spur tips, one spur was much longer than the other. We referred to these as primary and secondary spurs, respectively. The secondary spur generally merged with the primary spur beyond the end of the seed fibril to form a single tip projection.

### Molecular polarity and structure of fibril-spur variants

The observation of double spurs at the ends of seeds prompted us to survey the different forms of growth of the seed fibrils. Figure 4a shows a schematic representation of the results after 24 h of growth. Some generalisations were possible. For example, the most abundant structure (representing ∼ 50% of the sample) was a seed containing two spurs at the C end and one spur at the N end. Furthermore, in the 50 fibrils examined, seeds containing three or more spurs at one end, seeds lacking any spurs, and seeds with spurs located only at the N end were not seen.

### Mechanism of nucleation and growth from fibril seeds

The mechanism of spur formation from broken fibril ends should take into account the observation that the spur grows out of the blunt end whilst maintaining the *D* periodicity of the fibril, as well as explaining the observation of a primary (larger) and secondary (smaller) spur on the same fibril end. The simplest growth scheme for maintaining the *D* periodicity is shown in Fig. 4b, which shows a growth model that explains the continuity of the collagen staining pattern across the seed–spur junction and the mass distribution along the axis of the spur. The fact that the staining pattern is contiguous necessitates overlap of new molecules with those in the seed fibril. However, if the overlap with the seed fibril was limited to ∼ 1 molecular length, a pronounced bulge would be seen in the mass profile. The fact that the mass bulge does not occur and that there is an increase in mass per unit length along the whole length of the seed fibril indicate that the molecular addition extends along the entire length of the seed fibril, as shown in the lower diagram in Fig. 4b. This schematic figure shows the fibril seed broken at the C-telopeptide and an initial minimum overlap of the accreting molecules. Variants can be drawn with different possible breakage locations and different initial overlaps, but all would show the general feature of growth back over the seed fibril.

### Surface nucleation and propagation model of seed fibril growth

The observations described above support a simple model of surface nucleation and growth from the seed fibrils as shown schematically in Fig. 4c. The ends of the seed fibrils act as nucleation sites for spur growth (top diagram). The initial spurs grow bidirectionally (middle diagram) and the growth can propagate along the entire seed fibril (bottom diagram). Secondary spurs can form at a later stage, preferentially on the C end of the fibril (bottom diagram). This model is supported by STEM images of unstained fibrils that have loosened during grid preparation and show a continuity of microfibrillar substructure from the newly formed tip back along the surface of the seed fibril (Fig. 4d). The model permits a prediction of the increase in mass per unit length of the seed fibril relative to that of the new tip. The average ratio of the mass per unit length of the primary and secondary spurs close to the fibril seed–tip junction was measured from STEM images as 1:0.66. If the mass per unit length of the primary spurs is *m*_ℓ_ (as shown in Fig. 4c), then that of the secondary spur, *m*_ℓ_′, is 0.66 *m*_ℓ_ (using the experimental data). The increase in mass per unit length of the midsection of the seed fibril is then 2 *m*_ℓ_, and the mean mass per unit length of the spur extensions per seed fibril end close to the junction is (2 *m*_ℓ_ + 0.66 *m*_ℓ_)/2 = 1.3 *m*_ℓ_ for the three-spur form shown in Fig. 4a. This gives a value of 2 *m*_ℓ_/1.3 *m*_ℓ_ = 1.54 for the increase in mass per unit length of the seed fibril relative to that of the new tip. Calculating the appropriate ratio for each of the fibril forms shown in Fig. 4a and taking a weighted average according to the observed frequency of occurrence gives a predicted value of 1.48 for the mean increase in mass per unit length of the seed fibril relative to that of the tip. This is in approximate agreement to the experimental ratio of 1.40 (derived from the data shown in Fig. 2e). It is possible that some of the secondary spurs formed from primary spurs that had grown back along the seed fibril to the opposite end of the seed.

The proposed model does predict an axial region extending back along the seed fibril with increased mass per unit length (see first two stages in Fig. 4c). The experimental observation of this as a axial mass bulge is, however, dependent on the rate of axial growth back along the seed fibril and the sampling times of the experiment. If this axial growth rate was several times that of the axial extension rate of the spur into solution then an axial mass bulge would not be seen under the conditions of the experiment.

### Significance for tissue remodelling and repair

The observed surface nucleation and growth property of broken fibril ends has major implications for repair and remodelling processes in the extracellular matrix. The fibril growth behaviour observed in this study could serve as the basis for a process involving localised fibril breakage and regrowth of the fibril length fragments. This would be a far more efficient remodelling mechanism than wholesale replacement of the collagen fibril matrix. Despite the broken ends of the fibrils in this study having an abrupt fracture with a high proportion of broken collagen molecules they appear to be effective nucleation sites. We expect less abruptly broken fibril tips *in vivo* to also readily nucleate new growth. If regrowth of fragmented collagen fibrils occurs *in vivo*, for example, after tendon rupture or laceration, we might expect an increase in fibril number and increase in fibril diameter. Micromechanical damage occurs in tendon and ligament and leads to fatigue failure of these tissues when cyclically loaded after dissection,^13^ and this damage almost certainly involves collagen fibril breakage. The newly observed ability of fractured collagen fibrils to regrow, as reported in this communication, has relevance to tendon repair processes of both micro- and macrodamage events that occur in tissue and involve rupture of collagen fibrils.

## Figures and Tables

**Fig. 1:**
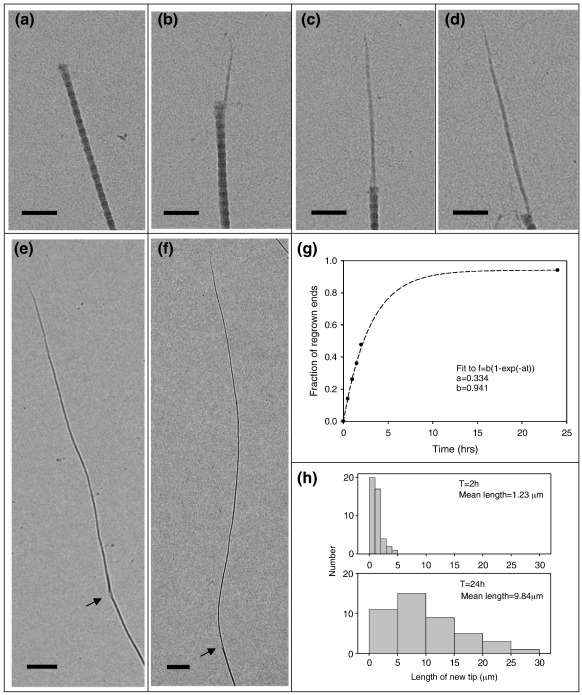
TEM of fibril seed growth after increasing incubation times in collagen solution. Fibril seeds were released from 13-day chick embryonic metatarsal tendon by crushing at liquid nitrogen temperature and then dispersing in “fibril dispersion” buffer [50 mM Tris–HCl, 50 mM EDTA (ethylenediaminetetraacetic acid), 150 mM NaCl, and 100 mM sucrose (pH 7.4)] in a Dounce homogeniser.^9^ The fibril suspension was diluted into a solution of acid-extracted type I collagen (50 μg/ml) at 34 °C in a Na_2_HPO_4_ (62 mM)/KH_2_PO_4_ (15 mM) buffer, *I* 0.2, pH 7.4, which had been set up according to the “warm-start” procedure.^10^ The extraction of type I collagen from bovine skin and subsequent purification of a monomeric solution was as described previously.^10^ Importantly the telopeptides (extrahelical domains) of the collagen molecule, known to have a critical role in fibril assembly, were preserved intact. A droplet of the seed suspension was placed on a carbon-filmed 200-mesh copper grid and left to adsorb for 1 min, washed with ultrapure water and air-dried. Samples shown were unstained and imaged in a Tecnai-12 transmission electron microscope (FEI, Eindhoven, the Netherlands). (a) Typical fibril length fragment released from 13-day chick embryonic tendon. (b) Projection formed on blunt fibril ends after 30 min and (c and d) after 2 h incubation in collagen solution. (e) and (f) show long tapered projections after 24 h incubation. The arrows indicate the junction of the new tip projections with the seed fibril. (g) Plot of fraction of regrown fibril ends against time. The points are shown fitted to a function of the form *f* = *b*(1 − exp(− *at*)), indicating an average nucleation half-time of 2.1 h and a fraction (6%) of fibril ends that are unable to nucleate fresh growth. (h) Projection length distributions for two incubation times (2  and 24 h). The data are consistent with a near-uniform axial growth rate for the fibril projections. Scale bars, (a)–(d), 0.25 μm; (e) and (f), 2 μm.

**Fig. 2:**
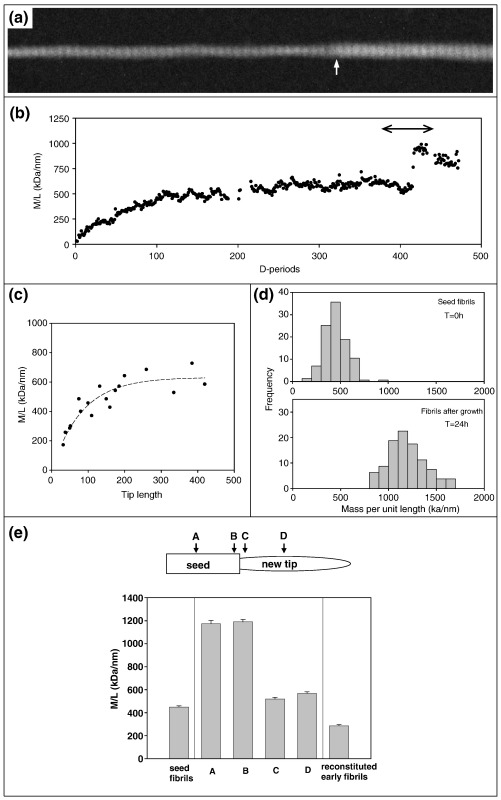
STEM data from seed fibrils after growth. Grid samples of unstained fibrils were prepared as described in the legend to Fig. 1. STEM imaging was on a Tecnai-12 TEM/STEM equipped with a high-angle annular dark-field detector (Fischione, Surrey, UK) with scan and image acquisition controlled by TIA software (FEI). Tobacco mosaic virus (TMV) was used as standard of mass per unit length (131 kDa/nm). (a) Annular dark-field STEM image of part of an unstained fibril tip formed after 24 h growth. The vertical arrow marks the junction of the seed fibril and the new tip projection. (b) Corresponding axial mass distribution measured from the STEM image in (a). The double-arrowed line corresponds to the axial region shown in (a). The plot is typical in showing an initial smooth increase in mass per unit length leading to a plateau region of limited mass per unit length. A plot of maximum mass per unit length *versus* projection length is shown in (c). The plot shows that a maximum lateral size of projections is attained after a length of ∼ 200 *D* periods (13.4 μm) has been reached. (d) Comparison of mass per unit length distribution of the initial seed fibrils with that of fibrils after 24 h of growth. (e) Mean values of mass per unit length for 50 seed ends with tip projections. The sites of measurement correspond to either side of the junction (B on seed, C on spur), midpoint along the seed fibril (position A) and the position of maximum mass per unit length along the spur (position D). These values are shown compared with the mean mass-per-unit-length values of the original seed fibrils and those of early fibrils reconstituted from the collagen solution at 200 μg/ml without seeds. On average, the fibril seeds show a 2.6-fold increase in mass per unit length after 24 h of growth. Error bars show SEM values.

**Fig. 3:**
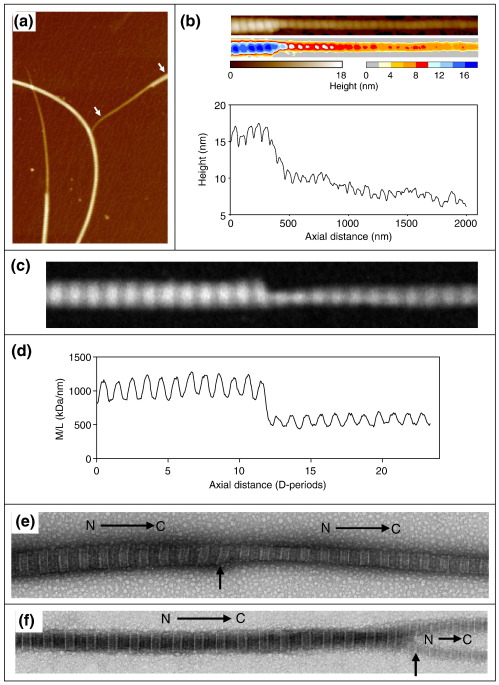
Ultrastructure over the growth junction of seed fibrils. AFM was performed on air-dried samples deposited on freshly cleaved mica using a Veeco Multimode with a Nanoscope IIIa controller operating in intermittent contact mode. (a) Typical AFM image of seed fibrils and tip projections after 2 h of growth. The specimen area is 4 μm × 6 μm and is scaled to 20 nm in height. A fibril plus tip lying between the white arrows in (a) is shown straightened (using ImageJ^11^) in the upper part of (b). The height scale is 18 nm and is shown displayed with continuous and discontinuous look-up tables. A height plot for a midposition along the fibril axis is shown in the lower part of (b). A dark-field STEM image of an unstained fibril seed–spur junction is shown in (c) and the derived axial mass distribution in (d). The periodic gap–overlap structure is clearly visible in both AFM and STEM and continues in phase over the junction between seed fibril and projection. (e and f) Typical TEM images of seed–spur junctions (vertical arrows) with 1 and 2 spurs, respectively, after negative staining with 2% uranyl acetate. Molecular polarity (N→C), as indicated by the stain pattern,^12^ is preserved across the junction as shown. *D* period = 67 nm.

**Fig. 4:**
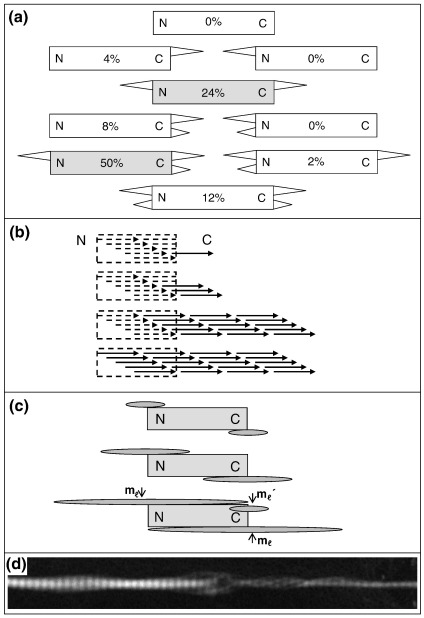
Alternative schemes for new tip growth on fibril ends. (a) Possible structural variants of fibril fragments having a primary spur or primary and secondary spurs on each end (N, C) of the fibril. Numbers show the observed frequency of each structural variant. Grey boxes show the two most abundant variants. (b) Model for the progressive addition of collagen molecules to a blunt fibril end whilst preserving the *nD* overlap between molecules, where *n* = 1 to 4. (c) Schematic representation of a simple nucleation and propagation growth model consistent with the observed growth forms and the mass per unit length data. The axial mass profiles of the new fibrillar growth (dark grey) will depend on the relative growth rates in solution and along the seed fibril surface. (d) STEM image of an unstained collagen seed fibril after 24 h of growth showing a continuity of microfibrillar substructure extending back from the newly formed tip along the surface of the seed fibril. *D* period = 67 nm.
